# Inclusion as illusion: erasing transgender women in research with MSM

**DOI:** 10.1002/jia2.25661

**Published:** 2021-01-26

**Authors:** Tonia C Poteat, L Leigh Ann van der Merwe, Jae Sevelius, JoAnne Keatley

**Affiliations:** ^1^ Department of Social Medicine University of North Carolina Chapel Hill NC USA; ^2^ Social, Health and Empowerment Feminist Collective of Transgender Women of Africa; ^3^ Department of Medicine University of California San Francisco CA USA; ^4^ Innovative Response Globally for Trans Women and HIV Oakland CA USA

**Keywords:** transgender women, sub‐Saharan Africa, HIV, key populations

We were excited to the see the publication of the *JIAS* Supplement entitled, “Engagement of African men and transgender women who have sex with men in HIV research.” We agree with the supplement editors that attention to these populations are long overdue [1]. The title of the supplement suggested that African transgender women would be highlighted in this special issue; and we were eager to read studies about this highly under‐served and under‐studied population. However, as we read each article, we were deeply disappointed to find that none of them focused exclusively or even primarily on transgender women.

For many years, transgender women have advocated to have their issues explored and understood separately from men who have sex with men (MSM) and other key populations. Transgender women’s social contexts are distinct from MSM. They face unique challenges when accessing HIV prevention and treatment, and meetings their needs requires focused research and programming. Each of us contributed to a *JIAS* special issue entitled, “HIV epidemics among transgender populations: the importance of a trans‐inclusive response” in 2016 [2]. In this issue, editors noted, “It cannot be overstated that transgender women are not MSM and should not be subsumed in a category that erases their identity [3].” Four years later, this special issue fails to heed that call.

In 2016, *Global Public Health* published a series of articles describing the impact of conflating transgender women with MSM. In that issue, Poteat et al. outline issues that may be completely missed when transgender women are assumed to have the same HIV risks as MSM [4]. Perez‐Brumer et al. identify how inclusion of HIV prevalence data from transgender women with MSM, artificially increases the reported prevalence among MSM as it simultaneously renders the elevated prevalence among transgender women invisible [5]. Kaplan et al. write to the limitations of Eurocentric notions of gender/sexuality and the impact on our understanding of drivers of HIV [6]. Sevelius et al. provide concrete examples from qualitative research with transgender women of the impact of conflation and erasure as a barrier to PrEP uptake among transgender women [7].

As HIV researchers who focus on the needs of transgender women in sub‐Saharan Africa, we know that such research is possible and that data from studies focused on African transgender women exist. A cursory review of recent published literature and abstracts from CROI 2020 and AIDS 2020 readily identified several research studies focused on transgender women in Africa [8, 9, 10, 11]. Meaningful inclusion of any population that appears in the title of a special issue should be a requisite. We urge *JIAS* to resist publishing studies that subsume transgender women within MSM‐focused research. Instead, we encourage *JIAS* to support the publication of studies that provide transgender women with the specific attention they deserve.

## COMPETING INTERESTS

All authors declare no conflicts of interest.

## AUTHORS’ CONTRIBUTIONS

TCP conceived of the manuscript and generated the initial draft. LLAVM, JS and JK reviewed and edited the draft. All authors have read and approved the final manuscript.

## AUTHORS’ INFORMATION

LLAVDM and JK are transgender community leaders who have engaged in research and advocacy to improve the health of transgender women for well over a decade. LLAVDM is based in South Africa where she leads an international organization for transgender women. JK is based in the United States where she worked in academic research and programme development for many years. Both LLAVDM and JK have generated original HIV research with transgender women as co‐principal investigators. TCP and JS have conducted community‐engaged research in partnership with transgender‐led organizations for more than a decade.
